# New approach for visualization of relationships between RR and JT intervals

**DOI:** 10.1371/journal.pone.0174279

**Published:** 2017-04-05

**Authors:** Pranas Ziaukas, Abdullah Alabdulgader, Alfonsas Vainoras, Zenonas Navickas, Minvydas Ragulskis

**Affiliations:** 1 Research Group for Mathematical and Numerical Analysis of Dynamical Systems, Kaunas University of Technology, Kaunas, Lithuania; 2 Prince Sultan Cardiac Center, Al Hasa, Al Hofuf, Saudi Arabia; 3 Institute of Cardiology, Lithuanian University of Health Sciences, Kaunas, Lithuania; Indiana University, UNITED STATES

## Abstract

This paper presents the concept of perfect matrices of Lagrange differences which are used to analyze relationships between RR and JT intervals during the bicycle ergometry exercise. The concept of the perfect matrix of Lagrange differences, its parameters, the construction of the load function and the corresponding optimization problem, the introduction of internal and external smoothing, embedding of the scalar parameter time series into the phase plane—all these computational techniques allow visualization of complex dynamical processes taking place in the cardiovascular system during the load and the recovery processes. Detailed analysis is performed with one person’s RR and JT records only—but the presented techniques open new possibilities for novel interpretation of the dynamics of the cardiovascular system.

## Introduction

ECG analysis is the basic, the primary, and the most studied noninvasive technique used for the contemporary investigation of the functionality of the cardiovascular system. The relevance of ECG parameters to the clinical practice is unquestionable ECG parameters are effectively used for the identification of various heart rate and conductivity defects, different heart hypertrophies and ischemic processes. Cardiac time intervals are sensitive markers of cardiac dysfunction, even when it goes unrecognized by conventional echocardiography [1]. There exist some opinions that diagnostic features of ECG parameters are completely understood—and that there is no need to seek for any new approaches in ECG analysis.

However, interrelations between EGC parameters is still an active area of research. A typical example is the relationship between RR and QT intervals. This relationship was first described by Bazzet [2] in a form of a functional correlation and the definition of tolerance intervals for QT. But one hundred years later, discussions about this relationship do not seem to be stopping. A number of new functional relations between these parameters were proposed. For example, functional relations among RR, JT, and QT intervals are evaluated in [3]. Many researchers were proposing new analytical expressions and confidence intervals for inter-parameter relationships—all adapted to their specific cohort [4–6]. Unfortunately, other researchers were not able to prove the validity of these relationships [7]. This is probably due to some of the reasons described below.

### Insufficient identification of the specificity of relationships between ECG parameters

For example, it is inappropriate to interpret QT as a characteristic of one physiological process. It is well known that QT is the sum of the depolarization and the repolarization of heart’s myocardium. However, depolarization and repolarization may have completely different variation tendencies. For example, depolarization and repolarization may both become shorter during the load—but sometimes depolarization becomes longer and repolarization becomes shorter (what is a rather common phenomenon during the extreme loads). Thus the sum of the depolarization and the repolarization would not represent a clearly determined physiological process during an exercise with a constantly increasing load—and a simple QT-RR relationship cannot describe the behavior of the human cardiovascular system in general. In other case, the examination of QT and JT intervals under the supervised consumption of dietary acids is presented in [8]. The correlation amongst QT, JT and their alternative counterparts is discussed in [9]. Similar problems exist for other relationships between ECG parameters (for example PQ-RR, etc.).

### A fixed model of relationships between ECG parameters cannot always hold even for a particular person

Relationships between ECG parameters do vary according to various physiological and pathological reasons. Holistic models interpret a human being as a complex system—where nonlinear chaotic processes play an important role in the relationships between different sub-systems and in generating reactions of these sub-systems [10]. Therefore, it is probably illogical to seek a unified deterministic model which could describe the relationships between ECG parameters. It makes sense to observe dynamical processes, dynamical relationships—which could exhibit complex chaotic behavior [11].

### Time-averaged relationships between ECG parameters are not able to represent the complexity of these relationships in different time scale lengths

The relationships between ECG parameters do depend on different time scales and other factors. For example, the influence of QRS duration on the JT and QT intervals is assessed in [12]. In addition, it is well known that there exist completely different short-term and long-term adaptations of the cardiovascular system to physical loads [11]. It becomes clear that the width of the observation window may seriously impede the computational results.

The complexity of the relationships, the chaotic nature of the processes, the fractality of time scales—all that yields a necessity to develop such computational techniques which could assess the dynamism of these relationships between RR and JT intervals. As mentioned previously, investigation of relationships between RR and JT intervals continues during the last decades. For example, this relationship is used for the detection of prolonged repolarization in ventricular conduction defects [13]—but the JT interval is constructed as a linear function of the RR interval in this study. Similarly, it is shown in [14] that heart rate-corrected JT interval is a good estimate of specific repolarization time in a cohort of physically fit university students.

On the contrary, the main objective of this paper is to propose a visualization technique of relationships between RR and JT intervals which could reveal the evolution of complex dynamical processes in the self-organization of the heart system during the load and the recovery processes.

## Methods

### Ethics statement

The research met all applicable standards for the ethics of experimentation. Permit to perform biomedical investigation was granted by Kaunas Regional Ethics Committee for Biomedical Investigations, No. BE-2-51, 23.12.2015. ECG bicycle ergometry exercise was used to record cardiac RR and JT intervals. Participants provided written informed consent prior to the experiment.

### Description

The assessment of functional ECG parameters was performed by using ECG analysis system “Kaunas-Load” [15–18] developed at the Institute of Cardiology, Lithuanian University of Health Sciences. The second ECG lead is used for the signal processing task. JT interval is evaluated as the time difference between the J point and the end of the T wave. The J point is defined as the moment where depolarization processes expire and repolarization processes begin. The J point is automatically detected by fixing the moment where the speed of ECG electrical processes change just before the repolarization. The end of the T wave is detected at the end of repolarization processes. This moment is identified by computing the intersection between the lead axis and the tangent line to the descending slope of the T wave. Numerous clinical trials were used to assess the measurement accuracy of the JT interval during the development of “Kaunas-Load” system [19].

**Fig 1:**
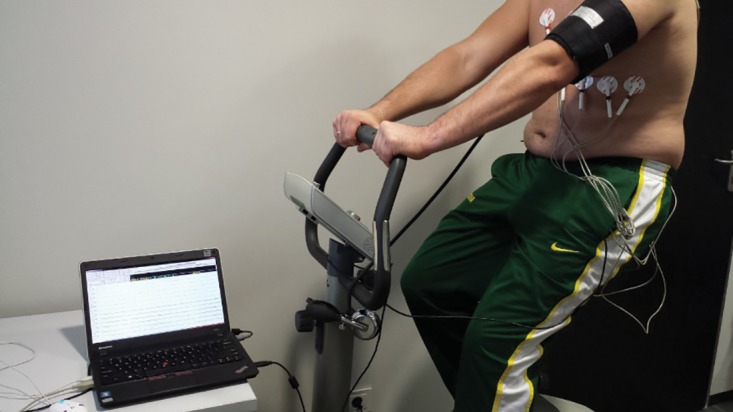
A general view of the experimental setup.

The bicycle ergometry system (Fig 1) is used for generating stepwise increasing physical loads—whlist “Kaunas-Load” is used for a synchronous registration of twelve different standard parameters of the ECG. The bicycle ergometry exercise is initiated at 50W load—and the load is increased by 50W every consecutive minute. The patient is asked to maintain a constant 60 revolutions per minute bicycle pedals spinning rate during the whole exercise. The load is increased up to 250W and the exercise is continued until the first clinical indications for the limitation of the load according to AHA (American Hearth Association) are observed. The cohort comprised 10 adult men within the age range of 29 and 36 years. All persons have been regular visitors of a local fitness club in Kaunas, and did not have any registered medical conditions.

Without losing the generality, we select two ECG parameters—RR and JT intervals. Sequences of RR and JT intervals are recorded during the bicycle ergometry experiment and denoted as vectors *x* = (*x*_1_, *x*_2_, …, *x*_*n*_) and *y* = (*y*_1_, *y*_2_, …, *y*_*n*_) accordingly.

## Perfect matrices of Lagrange differences

Given a scalar time series, derivatives of variables can be computationally assessed by Lagrange differences at the nodal point of the time series. It is well known that derivatives reconstructed from a scalar time series tend to intensify the noise embedded into that series. However, clever manipulations with derivatives may also help to detect and amplify small changes of the dynamical processes—which may not be visible in the original time series. Keeping this information in mind we use a combination of the scalar values of time series *x* and *y* (RR and JT intervals) and cross derivatives between elements of *x* and *y*. Our intention is to produce one single scalar attribute which could characterize dynamical relationships between *x* and *y*. We use square second order matrices, place different values and different differences as the elements of these matrices, and compute a single parameter representing a certain property of that matrix. The following sections are dedicated to the considerations on the architecture of these matrices and the selection of the parameter representing local relationships described by these matrices.

### Basic definitions

As mentioned previously, we will consider second order square matrices. We assume that every element of a matrix can be either a single element of the time series *x* or *y*—or a difference between elements of time series *x* and *y*:

$$\begin{array}{c} a\in \{\pm x,\pm y,\pm (x-y)\}. \end{array}$$ (1)

Note that symbols x and y in Eq (1) represent only the time series; indexes representing a particular time moment will be assigned later. Different signs can be assigned to elements of *x* and *y*. Also, differences are allowed only between elements of different time series (in order to minimize the straightforward amplification of noise). Second order square matrices comprising such elements are named as matrices of Lagrange differences (zeroth or the first order Lagrange cross differences).

#### Definition

A perfect matrix of Lagrange differences is a second order square matrix whereas elements of that matrix do satisfy the following requirements:

1. All elements of the matrix are different.
2. Zeroth order differences are located on the main diagonal.
3. First order differences are located on the secondary diagonal.
4. Indexes of *x* and *y* can take one of the three possible values: *i* ∈ {*n* − *δ*, *n*, *n* + *δ*}, where *n* is the current time moment and *δ* is the time lag; δ∈N.
5. The perfect matrix of Lagrange differences is lexicographically balanced—the number of symbols of *x* and *y* in the expressions of all elements of the matrix is the same.
6. The perfect matrix of Lagrange differences is balanced in respect of time—the number of indices with subscripts −*δ* and +*δ* in the expressions of all elements of the matrix is the same.

#### Example 1

Several examples of not perfect and perfect matrices of Lagrange differences.

$$\begin{array}{c} \left(\begin{array}{cc} 0 & x_{n+\delta }-y_{n+\delta }\\ x_{n-\delta }-y_{n-\delta } & y_{n} \end{array}\right)\;\text{- not a perfect matrix - the null element is not allowed.} \end{array}$$

$$\begin{array}{c} \left(\begin{array}{cc} y_{n} & x_{n+\delta }-y_{n+\delta }\\ x_{n-\delta }-y_{n-\delta } & y_{n} \end{array}\right)\;\text{- not a perfect matrix - no lexicographical balancing.} \end{array}$$

$$\begin{array}{c} \left(\begin{array}{cc} x_{n} & x_{n+\delta }-y_{n+\delta }\\ x_{n+\delta }-y_{n+\delta } & y_{n} \end{array}\right)\;\text{- not a perfect matrix - no balancing in respect of time.} \end{array}$$

$$\begin{array}{c} \left(\begin{array}{cc} x_{n} & x_{n+\delta }-y_{n+\delta }\\ x_{n-\delta }-y_{n-\delta } & y_{n} \end{array}\right)\;\text{- finally, this is a perfect matrix of Lagrange differences.} \end{array}$$

### The classification of perfect matrices of Lagrange differences

A natural question is about the number of different perfect matrices of Lagrange differences. In general we seek to represent six different elements of time series *x* and *y* as shown in Table 1.

**Table 1:** Six different elements of time series *x* and *y*.

*x*_*n*−*δ*_	*x*_*n*_	*x*_*n*+*δ*_
*y*_*n*−*δ*_	*y*_*n*_	*y*_*n*+*δ*_

These elements can be represented in a perfect matrix of Lagrange differences.

A graphical visualization of perfect matrices of Lagrange differences would help to classify these matrices and to interpret their structure. Zeroth order differences (±*x* and ±*y*) are located on the main diagonal—but indexes of these elements can be different. We draw circles around elements from Table 1 which are selected as zeroth order differences. For example, if ±*x*_*n*_ is selected as an element representing a zeroth order difference, then a circle is drawn around the element in the first row and the second column of Table 1.

A first order difference is visualized by drawing an arrow connecting the two elements of the difference. For example, an arrow connecting the element in the first row and the first column and the element in the second row and the first column is used to represent a difference ±(*x*_*n*−*δ*_ − *y*_*x*−*δ*_) in Table 1.

#### Example 2

**Fig 2:**
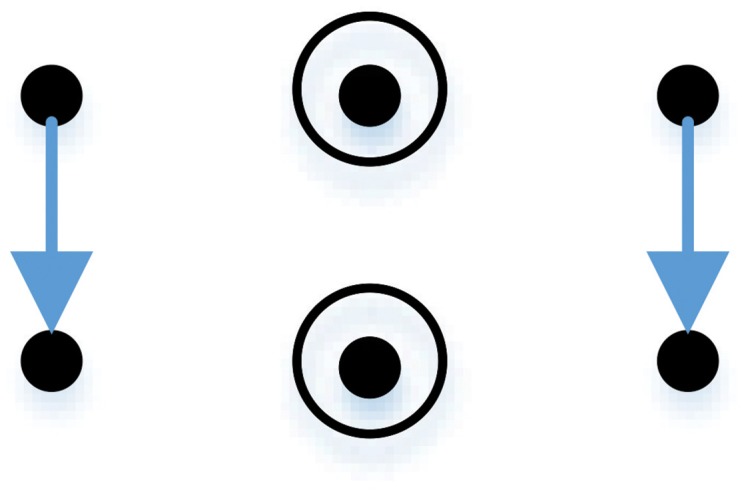
The graphical representation of a perfect matrix. This particular matrix is presented in Example 2.

As noted in Example 1, a matrix $L_{\delta ,n}^{\left(1\right)}=(\begin{array}{cc} x_{n} & x_{n+\delta }-y_{n+\delta }\\ x_{n-\delta }-y_{n-\delta } & y_{n} \end{array})\;$ is a perfect matrix of Lagrange differences. As previously, symbol *δ* denotes the time delay; *n*—the time moment around which the elements in Table 1 are centered around; (1) in the superscript denotes the number of the perfect matrices of Lagrange differences. The graphical representation of this matrix is shown in Fig 2.

**Fig 3:**
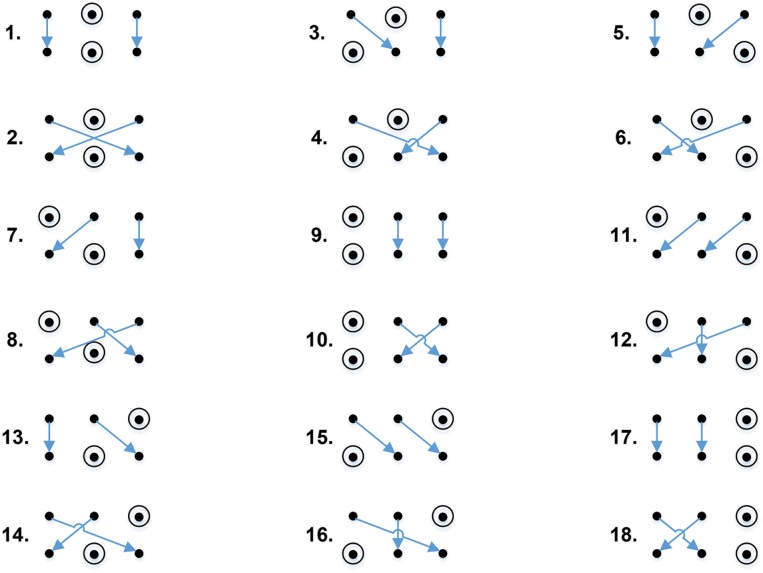
All possible graphical representations of perfect matrices of Lagrange differences.

Such graphical representation of perfect matrices of Lagrange differences allows an efficient classification of all possible perfect matrices. In fact, there are only 18 types of perfect matrices of Lagrange differences—all of them are illustrated in Fig 3.

One of the main characteristics of any square matrix are its eigenvalues (or the spectrum of the matrix). It is clear that elements on the diagonal and the anti-diagonal can be interchanged and their signs can be switched without affecting the maximal absolute eigenvalue. It means that every graphical representation of a matrix in Fig 2 can be a result of 2^4^ = 16 distinct perfect matrices of Lagrange differences in terms of the maximal absolute eigenvalue. Overall, 18 different representations yield 288 distinct perfect matrices of Lagrange differences.

### The construction of the optimization problem

As mentioned previously, a scalar parameter representing local relationships described by perfect Lagrange matrices must be selected. In general, the selection of such a parameter is an ill-posed inverse problem of parameter identification. Some sort of error function must be defined before different parameters could be assessed and compared in respect of their representativeness.

**Fig 4:**
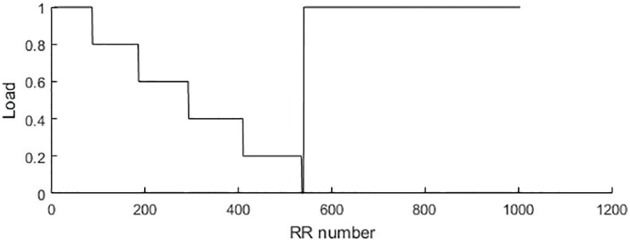
Load target function values for the person #1.

As mentioned in Section “Methods”, loads of the bicycle ergometry exercise are increased at every consecutive minute. However, the RR intervals do change during the exercise. The number of inter-beat intervals recorder during the first minute (before the exercise was started) and the further minutes are different (Fig 4). The *x*-axis in Fig 4 represents the index number of the *x*-time series. Note that the *x*-axis does not represent time—each inter-beat interval is different. Cumulative summation of the *x*-variable allows simple reconstruction of the time variable. However, we do not use the time variable in further computational experiments—all computations will be based on the *x*-time series data.

It is clear that Fig 4 is constructed for a single individual person. The investigated person did manage to reach 250W bicycle ergometry load—and then (almost immediately after reaching the highest load) the ergometry test was stopped. However, the recordings of the person’s ECG parameters continued during the recovery process.

Therefore, first 96 RR intervals fit into the first minute when the load was 0W; 108 RR intervals fit into the second minute (the first minute of the exercise when the load was 50W); 124 RR intervals into the 3-rd minute (100W load); 140 RR intervals into the 4-rd minute (150W load); 158 RR intervals into the 5-th minute (200W load) and only 5 RR intervals into the final part of the exercise when the load was increased to 250W (Fig 4). After the exercise was terminated, the recording of ECG parameters continued for another 5 minutes (Fig 4).

Moreover, we norm the opposite values of the load to the *y*-axis of Fig 4 and fit it to the interval [0, 1]. Therefore, 0W load is mapped to 1; 50W—to 0.8; 100W—to 0.6; 150W—to 0.4; 200W—to 0.2 and 250W—to 0 (Fig 4). In other words, we construct the target function—and the parameter representing local relationships described by perfect Lagrange matrices should follow this target function as close as possible.

We select the following parameters: the maximal absolute eigenvalue of a perfect matrix of Lagrange differences max |*λ*|, the minimal absolute eigenvalue min |*λ*|, the structural coefficient str = max |*λ*|/min |*λ*| and the discriminant dsk = (*a*_11_ − *a*_22_)^2^ + 4*a*_12_*a*_21_ (where indexes denote the location of elements). The time delay *δ* is set to 1.

Let us denote the scaled inverse values of the load (Fig 4) as *l*_*k*_; *k* = 1, 2, …, 1199. Now, the values of the particular parameter computed for the perfect matrix of Lagrange differences constructed from the RR (*x*-time series) and JT (*y*-time series) are denoted as *p*_*k*_; *k* = 1, 2, …, 1199 (note that *δ* = 1). The optimization problem is formulated as follows—minimize RMSE (root mean square error) between *l*_*k*_ and *p*_*k*_—by selecting the most appropriate parameter:

$$\begin{array}{c} \arg\min(\sqrt{\frac{1}{N}\sum_{k=1}^{N}{\left(l_{k}-p_{k}\right)}^{2}}); \end{array}$$ (2)

where the minimization is performed in respect of a particular expression of the parameter; values of *p*_*k*_ are normalized into interval [0, 1] before the optimization process is commenced.

Computational optimization of the best parameter is done by using ECG records of 10 persons. Every person did stop the bicycle ergometry exercise at different moments—so the number *N* in Eq (2) is different for each person (*N* = 1199 for the first person—his bicycle ergometry load protocol is shown in Fig 4). However, we do not preselect a single perfect matrix of Lagrange differences—we do average RSME for all 18 perfect matrices—the results are presented in Table 2. Note that the person whose load diagram is shown in Fig 4 is represented as Person #1 in Table 2.

**Table 2:** The averaged RMSE values for all perfect matrices.

Person	min |*λ*|	max |*λ*|	str	dsk
1	0.137	**0.085**	0.609	0.240
2	**0.294**	0.327	0.582	0.355
3	0.153	**0.134**	0.616	0.228
4	0.169	**0.105**	0.561	0.275
5	0.113	**0.095**	0.595	0.304
6	0.235	**0.123**	0.571	0.307
7	0.153	**0.114**	0.611	0.238
8	0.236	**0.204**	0.587	0.345
9	0.156	**0.132**	0.614	0.375
10	0.178	**0.089**	0.501	0.319

Here the parameter *δ* = 1 is fixed.

It can be seen that min |*λ*| is the best parameter for the second person—but max |*λ*| is the best parameter for all other persons. Therefore, we fix max |*λ*| as the best parameter representing the local relationships described by perfect Lagrange matrices.

### Internal and external smoothing

Computational experiments are continued with $\max|\lambda (L_{\delta ,k}^{\left(s\right)})|$ where $L_{\delta ,k}^{\left(s\right)}$ denotes a perfect matrix of Lagrange differences centered around time moment *k*. It is well known that moving averaging (MA) [20] helps to smooth coarse signals. Moreover, MA is frequently considered as a simple yet effective option for the prediction of irregular time series [21].

However, before dealing with MA, we introduce the procedure of internal smoothing. The radius of internal smoothing Δ defines how many parameter values computed for perfect matrices of Lagrange differences (at different *δ*) are averaged. For example, inner averaging (without external averaging) reads:

$$\begin{array}{c} \frac{1}{\Delta }\sum_{\delta =1}^{\Delta }\max\left|\lambda \left(L_{\delta ,k}^{\left(s\right)}\right)\right|. \end{array}$$ (3)

Now, the smoothing of the reconstructed parameter values at different time moments *k* is performed using the standard MA—such smoothing procedure is denoted as external smoothing. However, we do use only odd widths of the averaging windows (denoted as *m*) for MA—the averaging is constructed symmetrically around the time moment *k*. Let us denote the reconstructed parameter value as *p*_*k*_(*s*, Δ, *m*), where *k* is the time moment; Δ is the radius of internal smoothing; *m* is the radius of external smoothing. Then

$$\begin{array}{c} p_{k}\left(s,\Delta ,m\right)=\frac{1}{(2m+1)\Delta }\sum_{j=k-m}^{k+m}\sum_{\delta =1}^{\Delta }\max\left|\lambda \left(L_{\delta ,k}^{\left(s\right)}\right)\right|. \end{array}$$ (4)

Note that the order of internal and external averaging is not important (Eq (4)). However, that would not be the case if weights for different *δ* would be introduced for internal averaging (all weights set to be equal in this paper). Note that external averaging (without internal averaging) reads:

$$\begin{array}{c} p_{k}\left(s,1,m\right)=\frac{1}{2m+1}\sum_{j=k-m}^{k+m}\max\left|\lambda \left(L_{\delta ,k}^{\left(s\right)}\right)\right|; \end{array}$$ (5)

and the procedure of internal averaging (without external averaging) yields Eq (3).

#### Example 3

Let us consider the following time series: *x* = (*x*_1_, …, *x*_7_) = (1, 3, 2, 0, 4, 3, 1); *y* = (*y*_1_, …, *y*_7_) = (2, 1, 0, 4, 3, 1, 1) and let us compute *p*_4_(1, 2, 1).

**Fig 5:**
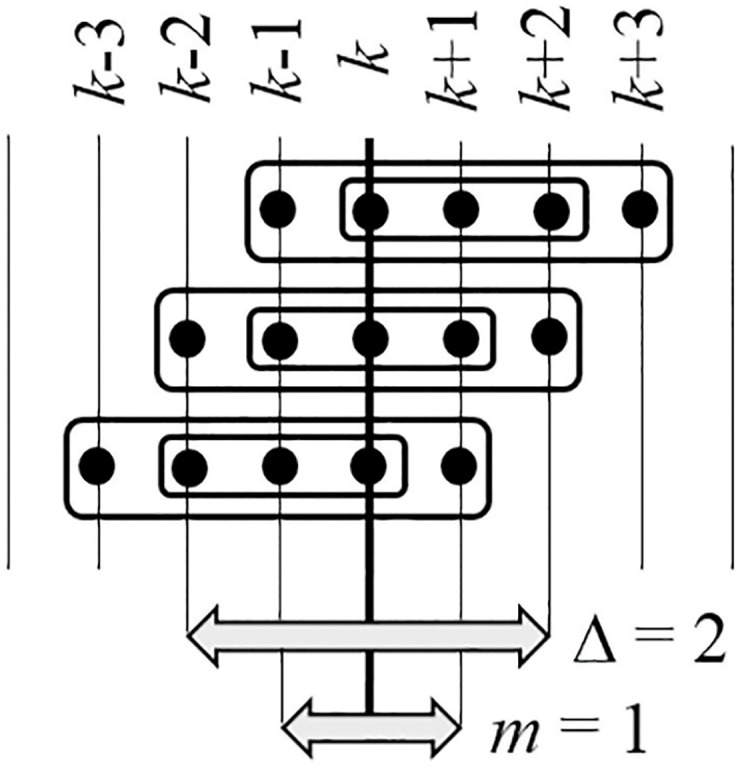
A schematic illustration of internal and external smoothing. Parameters Δ = 2 and *m* = 1.

A schematic diagram illustrating the computational procedures of internal and external smoothing is presented in Fig 5. First of all, the perfect matrix of Lagrange differences is constructed around *k* = 4: $L_{1,4}^{\left(1\right)}=(\begin{array}{cc} 0 & 4-3\\ 2-0 & 4 \end{array})$. The eigenvalues of $L_{1,4}^{\left(1\right)}$ are: *λ*_1_ ≈ −0.4495; *λ*_2_ ≈ 4.4495. Thus, $\max|\lambda (L_{1,4}^{\left(1\right)})|\approx 4.4495$.

Next, $L_{2,4}^{\left(1\right)}=(\begin{array}{cc} 0 & 3-1\\ 3-1 & 4 \end{array})$. The eigenvalues of $L_{2,4}^{\left(1\right)}$ are: *λ*_1_ ≈ −0.8284; *λ*_2_ ≈ 4.8284. Thus, $\max|\lambda (L_{2,4}^{\left(1\right)})|\approx 4.8284$.

Since Δ = 2, the procedure of internal smoothing yields: *p*_4_(1, 2, 0) ≈ 0.5(4.4495 + 4.8284) = 4.6389.

Since *m* = 1, computations must be repeated for *k* = 3 and *k* = 5. $L_{1,3}^{\left(1\right)}=(\begin{array}{cc} 2 & 0-4\\ 3-1 & 0 \end{array})$; *λ*_1,2_ ≈ 1 ± 2.6458*i*; $\max|\lambda (L_{1,3}^{\left(1\right)})|\approx 2.8284$. $L_{2,3}^{\left(1\right)}=(\begin{array}{cc} 2 & 4-3\\ 1-2 & 0 \end{array})$; *λ*_1,2_ = 1; $\max|\lambda (L_{2,3}^{\left(1\right)})|=1$. Now *p*_3_(1, 2, 0) ≈ 0.5(2.8284 + 1) = 1.9142.

$L_{1,5}^{\left(1\right)}=(\begin{array}{cc} 4 & 3-1\\ 0-4 & 3 \end{array})$; *λ*_1,2_ ≈ 3.5 ± 2.7839*i*; $\max|\lambda (L_{1,5}^{\left(1\right)})|\approx 4.4721$. $L_{2,5}^{\left(1\right)}=(\begin{array}{cc} 4 & 1-1\\ 2-0 & 3 \end{array})$; *λ*_1_ = 3; *λ*_2_ = 4; $\max|\lambda (L_{2,5}^{\left(1\right)})|=4$. Now *p*_5_(1, 2, 0) ≈ 0.5(4.4721 + 4) = 4.236.

Finally, $p_{4}\left(1,2,1\right)=\frac{1}{3}\left(p_{3}\left(1,2,0\right)+p_{4}\left(1,2,0\right)+p_{5}\left(1,2,0\right)\right)\approx 3.5964$

Now, it is possible to perform the minimization of RMSE in respect of *s* and Δ (Table 3). Note that this optimization is performed for the first person only (whose load diagram is presented in Fig 4). In order to lessen the extent of this computational experiment we set *m* = Δ and vary the internal and external smoothing from 1 to 8; the computation of RMSE is performed for all 18 perfect matrices of Lagrange differences (Table 3).

**Table 3:** RMSE values for person #1.

*s*, Δ	1	2	3	4	5	6	7	8
1	0.0948	0.0823	**0.0818**	0.0846	0.0848	0.0865	0.0891	0.0910
2	0.0907	0.0820	**0.0805**	0.0838	0.0870	0.0880	0.0910	0.0915
3	0.0907	0.0820	**0.0807**	0.0843	0.0866	0.0883	0.0908	0.0917
4	0.0953	0.0824	**0.0817**	0.0846	0.0860	0.0867	0.0896	0.0911
5	0.0920	0.0817	**0.0813**	0.0854	0.0888	0.0899	0.0926	0.0929
6	0.0922	0.0816	**0.0811**	0.0850	0.0873	0.0895	0.0922	0.0926
7	0.0904	0.0832	**0.0814**	0.0850	0.0881	0.0908	0.0912	0.0927
8	0.0905	0.0832	**0.0813**	0.0848	0.0877	0.0904	0.0909	0.0927
9	0.0946	0.0835	**0.0826**	0.0850	0.0879	0.0894	0.0902	0.0916
10	0.0947	0.0836	**0.0827**	0.0851	0.0881	0.0894	0.0902	0.0918
11	0.0918	0.0826	**0.0825**	0.0866	0.0896	0.0918	0.0918	0.0937
12	0.0917	0.0826	**0.0824**	0.0863	0.0893	0.0915	0.0914	0.0934
13	0.0898	**0.0819**	0.0828	0.0833	0.0853	0.0884	0.0892	0.0903
14	0.0896	**0.0821**	0.0827	0.0836	0.0856	0.0886	0.0894	0.0904
15	0.0945	**0.0821**	0.0825	0.0829	0.0853	0.0868	0.0881	0.0894
16	0.0947	**0.0822**	0.0829	0.0830	0.0855	0.0871	0.0883	0.0896
17	0.0909	**0.0817**	0.0850	0.0856	0.0882	0.0908	0.0908	0.0923
18	0.0911	**0.0818**	0.0845	0.0859	0.0885	0.0909	0.0910	0.0924
Average RMSE	0.0922	0.0824	**0.0822**	0.0847	0.0872	0.0892	0.0904	0.0917

Different matrices *s* and parameters Δ are presented in rows and columns respectively.

It can be observed that a moderate internal and external smoothing (*m* = Δ = 3) helps to minimize RMSE for almost all perfect matrices of Lagrange differences. In fact, the average value of RMSE (averaged for *s* = 1, 2, …, 18) is minimal at *m* = Δ = 3 (Table 3). Therefore, we will fix *m* = Δ = 3 for further computations.

Also, it can be observed that the RMSE value has a lower dependency on *s* than Δ (Table 3). Apparently, effects induced by the smoothing operation have a greater impact to the RMSE than the parameter *s* (at a fixed smoothing window). Some minimal smoothing helps to remove the noise and to minimize the RMSE (at Δ = 3). However, wider smoothing windows distort the signal [22] (the signal loses the variability, the effect of a time delay is introduced).

## Visualization of the load and the recovery processes

**Fig 6:**
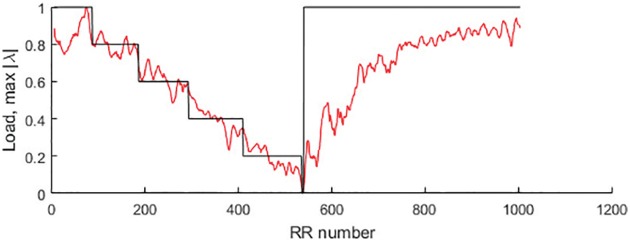
Load target function and optimal parameter values for the person #1.

Table 4 shows that the lowest RMSE value is achieved at *m* = Δ = 3 and *s* = 3 (the third perfect matrix of Lagrange differences). The variation of *p*_*k*_(3, 3, 3) (normalized into interval [0, 1]) is illustrated in Fig 6.

**Table 4:** RMSE values.

*s*, Person	1	2	3	4	5	6	7	8	9	10
1	0.082	0.322	0.119	0.109	0.094	0.126	0.112	0.219	0.124	0.088
2	0.081	0.324	0.119	0.103	0.095	0.125	0.112	0.221	0.123	0.088
3	**0.081**	0.318	0.118	0.107	0.095	0.114	0.112	0.203	0.118	0.088
4	0.082	0.317	0.118	0.105	0.096	0.121	0.113	0.204	**0.118**	0.088
5	0.081	0.325	0.118	0.105	0.095	0.111	0.112	0.208	0.121	**0.087**
6	0.081	0.325	0.119	0.101	0.095	0.105	0.112	0.208	0.120	0.087
7	0.081	0.317	0.119	0.105	0.099	0.118	**0.111**	0.224	0.130	0.096
8	0.081	0.318	0.119	**0.101**	0.099	0.127	0.112	0.225	0.126	0.096
9	0.083	**0.315**	0.118	0.106	0.101	0.109	0.112	0.208	0.128	0.096
10	0.083	0.316	0.118	0.102	0.103	0.136	0.113	0.208	0.125	0.096
11	0.083	0.321	0.117	0.102	0.102	0.120	0.112	0.211	0.127	0.097
12	0.082	0.321	**0.117**	0.102	0.101	**0.102**	0.112	0.210	0.127	0.098
13	0.083	0.323	0.133	0.112	**0.093**	0.140	0.113	0.215	0.134	0.090
14	0.083	0.324	0.134	0.109	0.094	0.138	0.113	0.217	0.133	0.089
15	0.083	0.322	0.136	0.110	0.095	0.134	0.113	0.200	0.130	0.089
16	0.083	0.321	0.136	0.109	0.095	0.137	0.113	**0.200**	0.130	0.088
17	0.085	0.326	0.134	0.106	0.095	0.124	0.114	0.203	0.132	0.090
18	0.085	0.327	0.134	0.104	0.095	0.122	0.114	0.204	0.131	0.088

The parameters are *m* = Δ = 3, different matrices *s* (rows) and persons 1 to 10 (columns).

Fig 6 shows the variation of the optimal parameter *p*_*k*_, reconstructed using the optimal matrix of Lagrange differences and optimal smoothing. Yet, Fig 6 reveals interesting features. It is well known that the “collapse of complexity” happens with the heartbeat time series at different pathologies [23, 24]. The parameter *p*_*k*_ reaches zero at the moment when person #1 cannot continue the bicycle ergometry test at the maximal load. But *p*_*k*_ is the modulus of the maximal eigenvalue of the perfect matrix of Lagrange differences. The complexity of the system is minimal when the modulus of the maximal eigenvalue of the matrix of differences becomes minimal. That also explains why the load diagram (Fig 4) represents the scaled inverse values of the actual load.

The variation of the parameter *p*_*k*_ in Fig 6 also reveals an interesting phenomenon. The system (represented by *p*_*k*_) tries to stabilize around the scaled normalized constant value of the load—that is particularly clear around the load level 0.8 in Fig 6. This constant value of the load can be interpreted as a stable fixed point—which losses its stability due to the fatigue. Similar loss of stability in a quasi-isometric arm-curl exercise reflected by the increase of low-frequency fluctuations is observed in [25].

**Fig 7:**
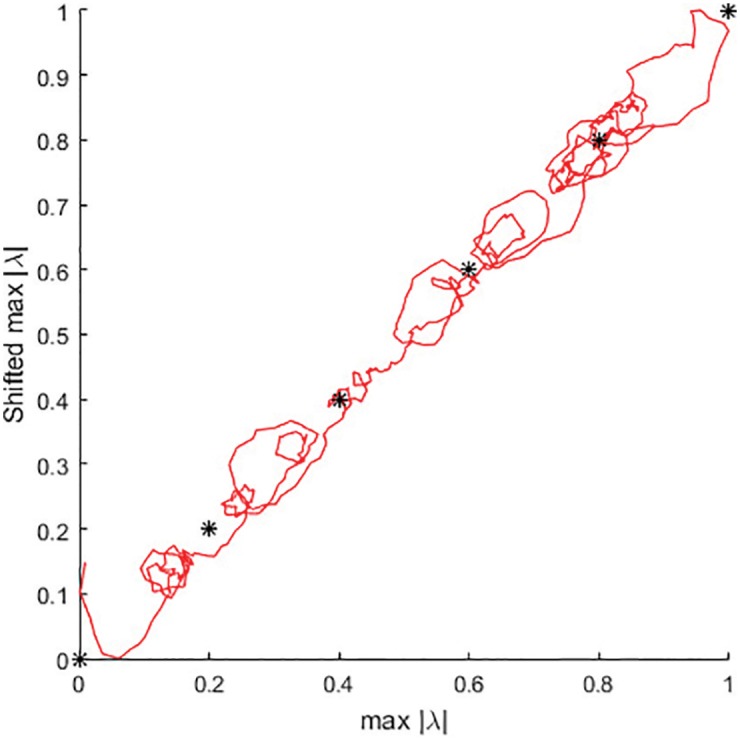
Phase plane of the optimal parameter series *p*_*k*_ during the load. The time delay is *τ* = 4.

It is well known that phase maps of bioelectrical signals my useful for the investigation of dynamical processes [26]. In order to better represent the attractors (and to assess their stability) we also visualize the dynamical processes of Fig 6 in a phase plane. Time-delay embedding [27] is used to visualize temporary stabilization and the subsequent loss of the stability of attractors in the phase plane (Fig 7); the optimal time delay *τ* is determined by using the technique presented in [28] (*τ* = 4 RR intervals). The normalized loads at 1, 0.8, 0.6, 0.4, 0.2 and 0 are shown as star type markers in Fig 7.

Temporal stabilization of *p*_*k*_ around 0.8 fixed-point attractor is especially well expressed in Fig 7. It is clear that person #1 would be able to continue the bicycle ergometry test for a much longer time at the load equal to 0.8. However, the increased load (to 0.6) forces the system to re-organize (Fig 7). Note that these reorganization processes are interpreted and visualized just by using two time series—RR and JT interval sequences.

The reorganization process to the 0.6 fixed-point attractor is rather complex. Initially, the system tries to loop around a non-existing attractor slightly higher than 0.6—then goes through some transients—and settles around 0.6 (Fig 7). Again, it is clear that person #1 could continue the exercise much longer at the load 0.6.

However, the load is again increased to 0.4. Now, the system quickly converges to the 0.4 fixed-point attractor—and the complexity of the transient processes is much lower compared to the previous transitions (Fig 7). Again, person #1 could continue the exercise for a longer time than he is allowed.

But the convergence of the process to 0.2 fixed-point attractor is much more complicated—in fact the sequence of *p*_*k*_ never stabilizes around 0.2 (Fig 7). It seems that person #1 would not be able to continue the load at 0.2 much longer than he already did. The transition to the 0 fixed-point attractor never happens. Instead “the collapse of complexity” happens and person #1 terminates the bicycle ergometry test.

**Fig 8:**
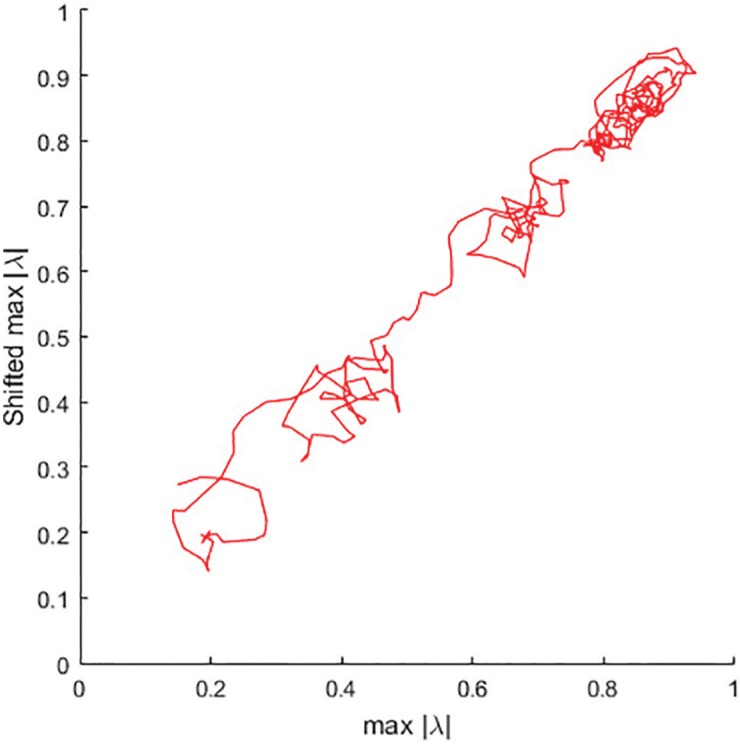
Phase plane of the optimal parameter series *p*_*k*_ after the load. The time delay is *τ* = 4.

It is equally interesting to observe the recovery processes right after the bicycle ergometry test is terminated. The inverse effect of the “collapse of complexity” is observed in Fig 8. The trajectory not only moves away from the point of collapse—the complexity of the trajectory itself is drastically changes over time.

Initially, the trajectory rapidly moves towards a temporarily stable attractor around 0.45 (Fig 8). However, this attractor loses the stability as the recovery processes continue. Finally, the system’s trajectory plots a chaotic attractor around 0.8. Such chaotic behavior of the heart could be considered as the validation of the presented model—since it is well known that heart is a chaotic system [29, 30].

## Further discussions and concluding remarks

A computational technique for visualization of complex transient processes of the heart parameters (RR and JT) is developed in this paper. It is based on the extensive application of novel algorithms and concepts originating from the broad field of nonlinear systems science and engineering. A natural question is whether such complex computations are required—maybe straightforward visualization of RR and JJ in a phase plane would reveal similar relationships?

**Fig 9:**
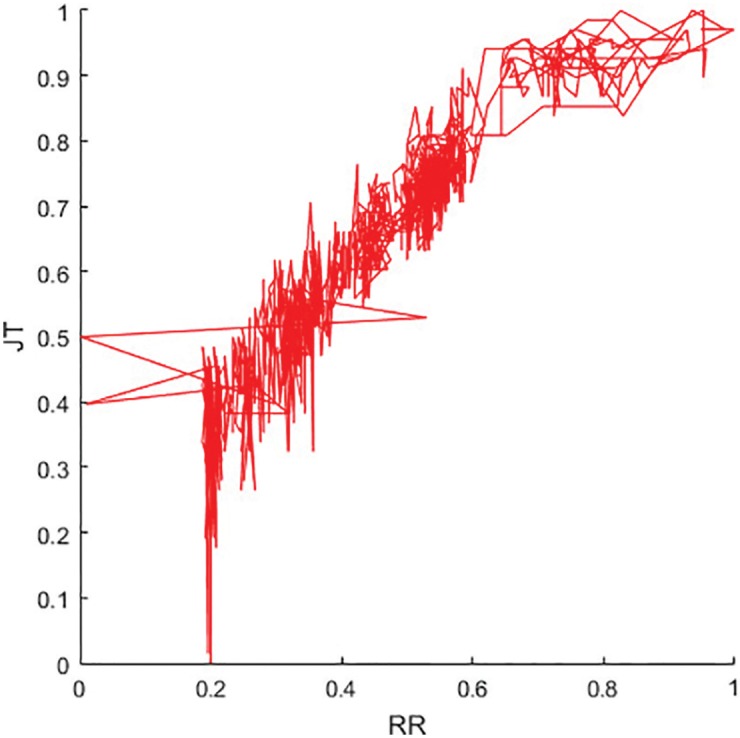
Phase plane of the RR and JT interval series.

The variation of RR—JT intervals during the bicycle ergometry test (for the first person) is visualized in Fig 9. Unfortunately, Fig 9 reveals essentially only a single relationship—RR (and JT) intervals do become shorter at the load.

This paper focuses on the development of a novel visualization technique of relationships between RR and JT intervals. But it is unclear if similar information on the self-organization of the heart system during the load and the recovery could not be retrieved from other cardiac intervals (for example RR and QRS; RR and QT). However, it appears that RR and QRS, RR and QT intervals yield completely different representations (S2 Appendix).

An important aspect of the presented computational technique is that an optimal set of parameters (*s*, *m* and Δ) is reconstructed for every individual person based on his RR, JT time series and the load data. The resulting optimal time series *p*(*s*, *m*, Δ) minimizes the individual load target function and is used for visual characterization of the self-organization of the heart system.

From the computational point of view, it would be possible to further minimize the differences between *p*(*s*, *m*, Δ) and the load target function by selecting optimal parameters *s*, *m* and Δ for each individual load step (not for the whole load history data). However, such an approach is not used due to two important reasons.

Firstly, the optimal time series *p*(*s*, *m*, Δ) would be a discontinuous function (at the time moments when the load is changed). That would complicate the interpretation of the graphical phase diagrams. Secondly, it would be unclear which parameter set should be used for the recovery. In other words, such splitting of the optimization procedure would not allow to visualize the recovery processes—which are considered to be equally (or even more) important than the load processes [31].

Another important aspect of this study is that we have identified the best fitting perfect matrix of Lagrange differences for the first person (*s* = 3). We do speculate that the particular number of the perfect matrix of Lagrange differences may serve as an identifier of dynamical processes taking place in the heart system during load and recovery.

The following computational experiment is performed for the illustration of this hypothesis. We use the same parameter (the maximum absolute value of the eigenvalues of a perfect matrix of Lagrange differences), the internal and the external smoothing (*m* = Δ = 3)—but instead of fixing the third matrix of Lagrange differences (*s* = 3) we perform the minimization of RMSE for every person for every possible perfect matrix of Lagrange differences (Table 4).

It is interesting to note that RMSE values for the second person (for all *s* values) are more than three time higher compared to the RMSE values for the first person (Table 4). But the same effect can be observed in Table 2 and could be probably explained by a guess that the self-organization of the heart system for the second person does function differently.

Nevertheless, it is possible to reconstruct a best fitting perfect matrix of Lagrange differences for every single person (Table 4; S1 Appendix). As mentioned previously, we hypothesize that the sequential number of this matrix is a specific identifier of the dynamical relationships and self-organization taking place in a particular person’s heart system.

However, the proposed technique for visualization of relationships between RR and JT intervals does not propose a single marker which can detect such conditions as atrial fibrillation, supraventricular tachycardia, or sudden cardiac death. Instead, we propose a computational technique which could reveal the complexity of the self-organization of the heart system during the load and the recovery processes. This complexity is represented by the parameter *p*(*s*, *m*, Δ). A physician can observe the “collapse of complexity” at the end of the bicycle stress test, temporary stabilization of transient attractors during the load, rich dynamical behavior of the heart system during the recovery process.

We doubt if it is possible to quantity such complex transient dynamics by a single biomarker. However, development of pattern classification algorithms for automatic analysis of transient orbits generated by *p*(*s*, *m*, Δ) (and relating these patterns to novel markers for early disease diagnosis) remains a definite objective of future research.

## Supporting information

S1 Appendix Detailed analysis for persons #2 − 5. Target load functions, phase planes during the load and after the load for persons #2, #3, #4 and #5.
(PDF)

S2 Appendix Analysis using RR, QRS, QT intervals. Other cardiac intervals yield different representation of the self-organizational processes.
(PDF)

S1 Fig Visual results for persons #2 − 5. Target load functions, phase planes during the load and after the load for persons #2 (parts a, b, c), #3 (parts d, e, f), #4 (parts g, h, i) and #5 (j, k, l).
(TIF)

S2 Fig Visual analysis using RR-QRS and RR-QT intervals. Using RR-QRS intervals: target load function and *p*_*k*_(2, 3, 3) (part a), phase plane during the load (part b), after the load (part c), and straightforward mapping RR-QRS (part d) for person #1. Using RR-QT intervals: target load function and *p*_*k*_(9, 3, 3) (part e), phase plane during the load (part f), after the load (part g), and straightforward mapping RR-QT (part h) for person #1.
(TIF)

S1 Data Original data files. The supporting information files include unprocessed data from ECG analysis system “Kaunas-Load” for all subjects who were part of this research.
(ZIP)
