# Supplementary material for: New approach for visualization of relationships between RR and JT intervals
Source: PLoS One. 2017 Apr 5;12(4):e0174279. doi: 10.1371/journal.pone.0174279 (PMC5381794; doi:10.1371/journal.pone.0174279)
Supplement: S1 Appendix — Target load functions, phase planes during the load and after the load for persons #2, #3, #4 and #5. (PDF) [file pone.0174279.s001.pdf]

S1 Appendix. Detailed analysis for persons #2 − 5**.** Target load functions, phase planes during the load and after the load for persons #2, #3, #4 and #5.

Person #2. RMSE for person #2 is highest among all persons and is almost 4 times higher compared to person #1 (Table 4). Moreover, the optimal parameter for person #2 is min $|\lambda|$, not max $|\lambda|$ (Table 2). Nevertheless, as discussed previously, we keep the parameter max $|\lambda|$ for person #2.

It is clear that the self-organization of the heart system of person #2 is different from all other persons - $p_k(9, 3, 3)$ is fluctuating around 0.5 before the exercise (S1 Fig part a). The "collapse of complexity" is also observed at the end of the bicycle ergometry exercise (S1 Fig part b) - but the recovery process is different compared to all other persons. At the beginning of the recovery process, the heart system fluctuates widely in the phase space (S1 Fig part c). Such fluctuations could be explained by the competition between two competing attractors - one at $(1, 1)$ and the other at $(0.5, 0.5)$. Finally, attractor $(1, 1)$ loses stability and the system converges to attractor $(0.5, 0.5)$. Also, person #2 terminated the exercise much earlier compared to other persons. We speculate that such untypical behavior of person #2 is associated to some illness or at least some sort of disorder in the self-organization of his heart system.

Person #3. The self-organization of the heart system of person #3 (S1 Fig parts d, e and f) is much more similar to person #1 than to person #2. However, there are some important differences. First of all, $s = 12$ for person #3. Secondly, the recovery process of person #3 is less monotonic if compared to person #1, #4 and #5. An abrupt jump of the heart system to the state of rest can be observed at around $RR = 950$ (S1 Fig part f). But it appears that this transition was too fast - and the heart system quickly re-organizes itself towards a slow recovery. A similar "pre-mature" peak of $p_k(12, 3, 3)$ can be observed at around $RR = 1050$. Finally, the process slowly converges to the chaotic state of rest.

Person #4. The dynamics of the self-organization of person #4 is also rather unique (S1 Fig parts g, h and i). The general trend of the self-organization during the load is similar to other persons (except person #2). However, the variability of $p_k(8, 3, 3)$ is much higher near the point of collapse (compared to persons #1 and #3). On the other hand, the recovery process after the load is much slower and much less turbulent compared to persons #1 and #3 (S1 Fig part i). It is clear that the length of the record was too short for person #4 to return to the state of rest.

Person #5. The self-organization of the heart system of person #5 (S1 Fig parts j, k and l) is almost identical to person #4. The main observable difference between persons #5 and #4 is in the middle region of the load around $200 < RR < 400$ where the variability of $p_k(13, 3, 3)$ is much higher.

Concluding discussion for S1 Appendix. The proposed computational technique is used to visualize self-organization processes for different persons during the load and the recovery. Visual interpretation of relationships between RR and JT intervals helps to reveal many interesting features. And though the general effect of the "collapse of complexity" at maximal loads is present for all persons, individual peculiarities of the transient processes (especially during the recovery) are strikingly different. These individual variations can be used to characterize the intrinsic individual dynamics of each person. Such individual qualitative differences do confirm that every person is unique. A computational technique being able to determine and to visualize this individual uniqueness is the main result of this paper.
