# Supplementary material for: New approach for visualization of relationships between RR and JT intervals
Source: PLoS One. 2017 Apr 5;12(4):e0174279. doi: 10.1371/journal.pone.0174279 (PMC5381794; doi:10.1371/journal.pone.0174279)
Supplement: S2 Appendix — Other cardiac intervals yield different representation of the self-organizational processes. (PDF) [file pone.0174279.s002.pdf]

S2 Appendix. Analysis using RR, QRS, QT intervals. Other cardiac intervals yield different representation of the self-organizational processes.

Relationships between RR and QRS intervals. The target load function and the sequence $p_k(2,3,3)$ is illustrated in S2 Fig part a (the optimal $s = 2$; $RMSE = 0.427$). It is clear that even the optimal sequence $p_k(2,3,3)$ cannot approximate the target load function. Instead of decreasing during the load, the trend of $p_k(2,3,3)$ is clearly increasing (S2 Fig part a). Then, an instantaneous "collapse of complexity" happens with a time delay after the termination of the bicycle stress test. Finally, $p_k(2,3,3)$ decreases during the recovery process (S2 Fig part a).

It is clear that the relationship between RR and QRS intervals reveals completely different aspects of the self-organization of the heart system. A completely new strategy for the construction of the target load function is required in order to interpret this relationship.

Relationships between RR and QT intervals. Relationships between RR and QT intervals are visualized in S2 Fig parts e-h. Note that QT = JT + QRS. The variation of $p_k(9,3,3)$ is shown in S2 Fig part e (it inherits properties from both JT and QRS dynamics); $RMSE = 0.285$. Analogously, the relationship between RR and QT intervals reveals different aspects of the self-organization of the heart system and a different strategy is required for the construction of the target load function.

Concluding discussion for S2 Appendix. Computational visualization of relationships between RR-QRS, RR-QT intervals is performed using unmodified algorithm employed for visualization of relationships between RR and JT intervals. It is completely clear that the target load function (and the whole optimization process) should be modified. Relationships between RR-QRS, RR-QT intervals yield different information on the dynamics of the heart system. Computational visualization of these relationships falls out of the scope of this paper and is a clear objective of future research.
