# Supplementary figures and images for: New approach for visualization of relationships between RR and JT intervals
Source: PLoS One. 2017 Apr 5;12(4):e0174279. doi: 10.1371/journal.pone.0174279 (PMC5381794; doi:10.1371/journal.pone.0174279)

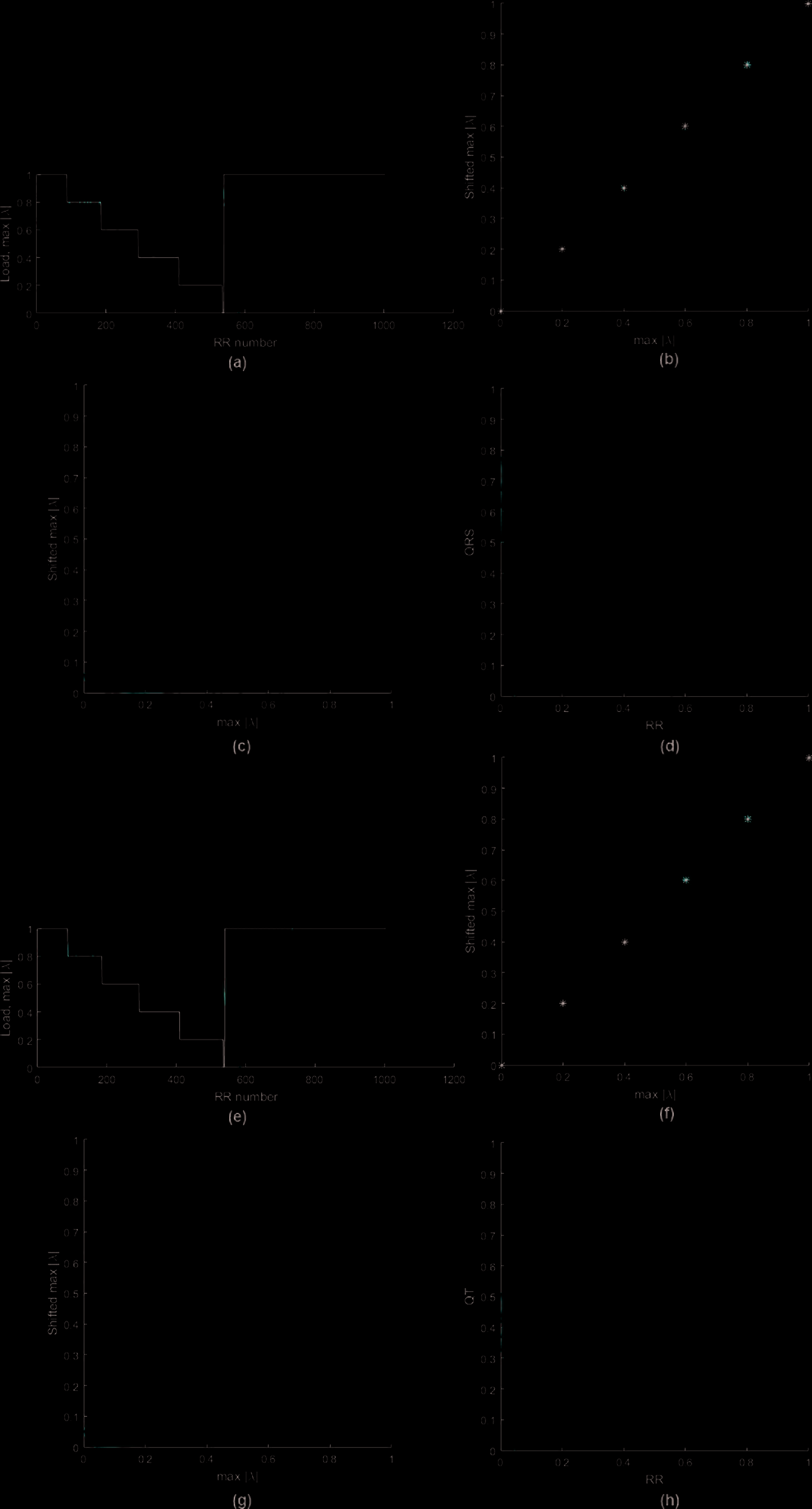

Supplement: S2 Fig — Using RR-QRS intervals: target load function and pk(2, 3, 3) (part a), phase plane during the load (part b), after the load (part c), and straightforward mapping RR-QRS (part d) for person #1. Using RR-QT intervals: target load function and pk(9, 3, 3) (part e), phase plane during the load (part f), after the load (part g), and straightforward mapping RR-QT (part h) for person #1. (TIF) [file pone.0174279.s004.tif]
